# Supplementary figures and images for: Daily replacement of very high-fat diet stabilizes food intake and improves mouse welfare by ensuring food quality
Source: PLoS One. 2023 Sep 18;18(9):e0291347. doi: 10.1371/journal.pone.0291347 (PMC10506720; doi:10.1371/journal.pone.0291347)

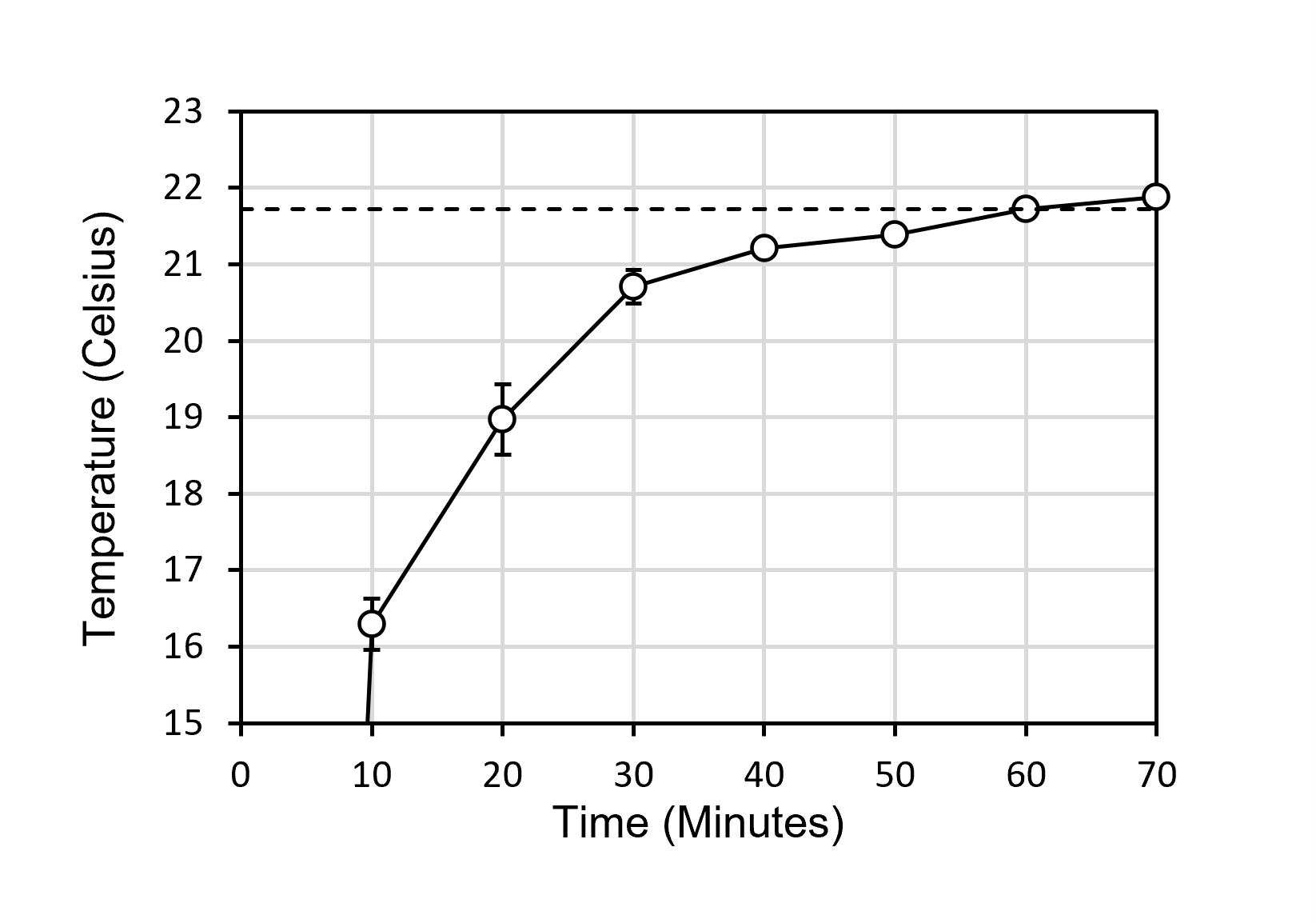

Supplement: S1 Fig — VHFD removed from storage at -20°C returns to room temperature (21.7°C; dotted line) after ∼60 minutes. All values represent AVG ± SEM. (TIF) [file pone.0291347.s001.tif]

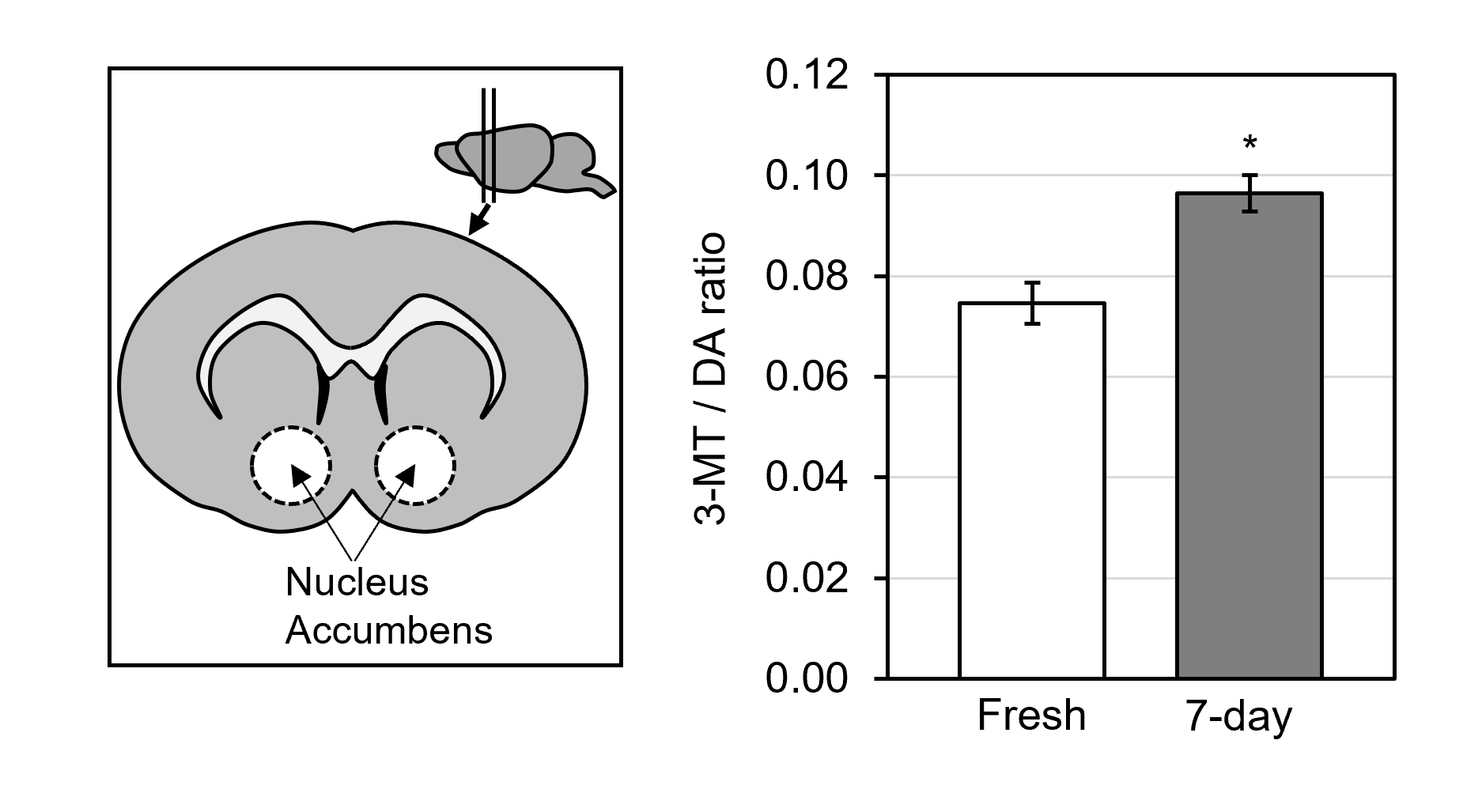

Supplement: S2 Fig — The 3-MT / DA ratio, indicative of dopamine release, was significantly higher in the nucleus accumbens after consuming 7-day-old VHFD compared to freshly thawed VHFD. [Independent samples T-test: t(6) = -3.98, p = 0.007, d = -2.81] All values represent AVG ± SEM. (TIF) [file pone.0291347.s002.tif]

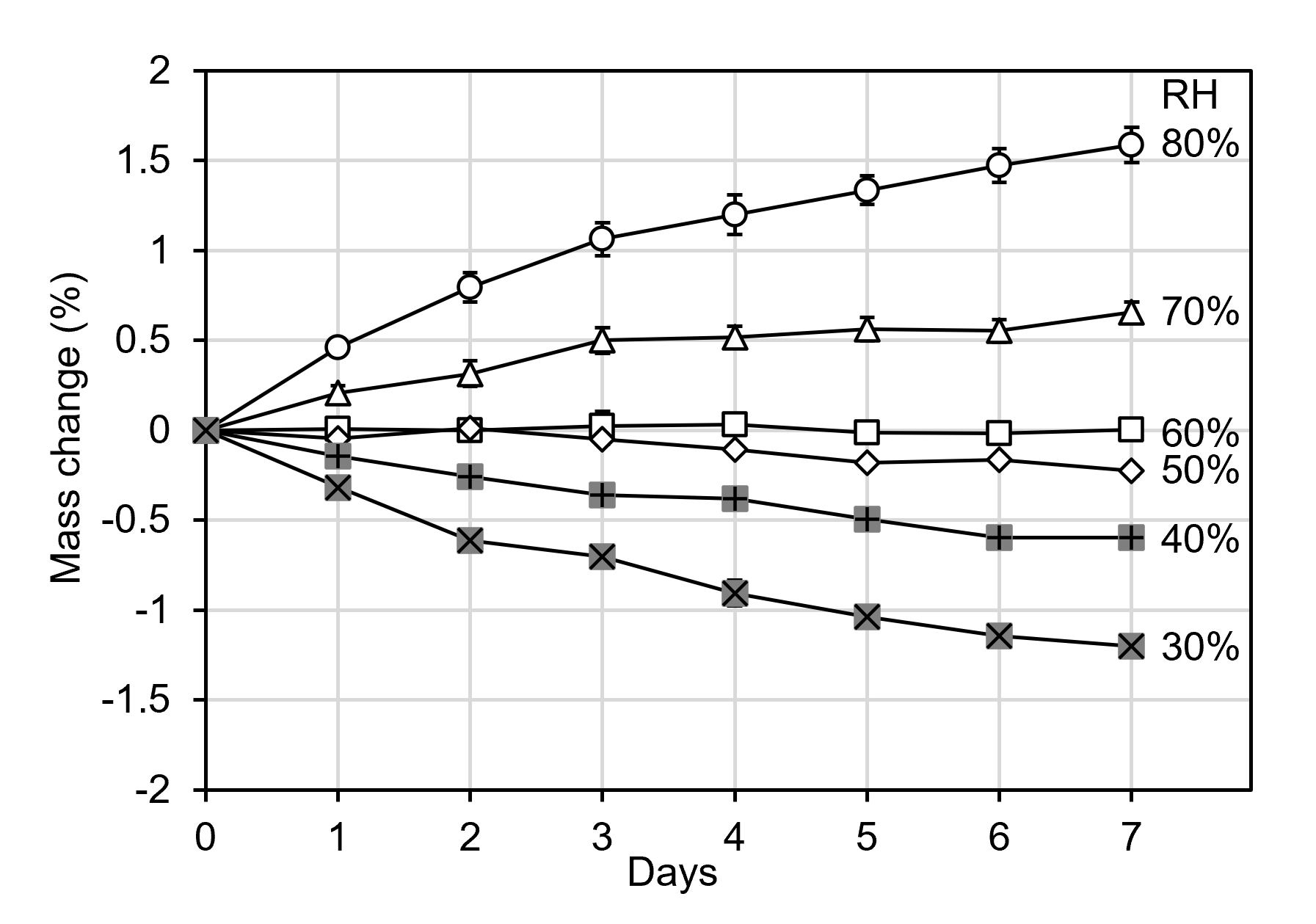

Supplement: S3 Fig — VHFD stored at a different relative humidity (RH) gained or lost a different amount of mass over 7-days. [ANOVA F(5,30) = 183, p < 0.001, ηp2 = 0.968] All values represent AVG ± SEM. (TIF) [file pone.0291347.s003.tif]

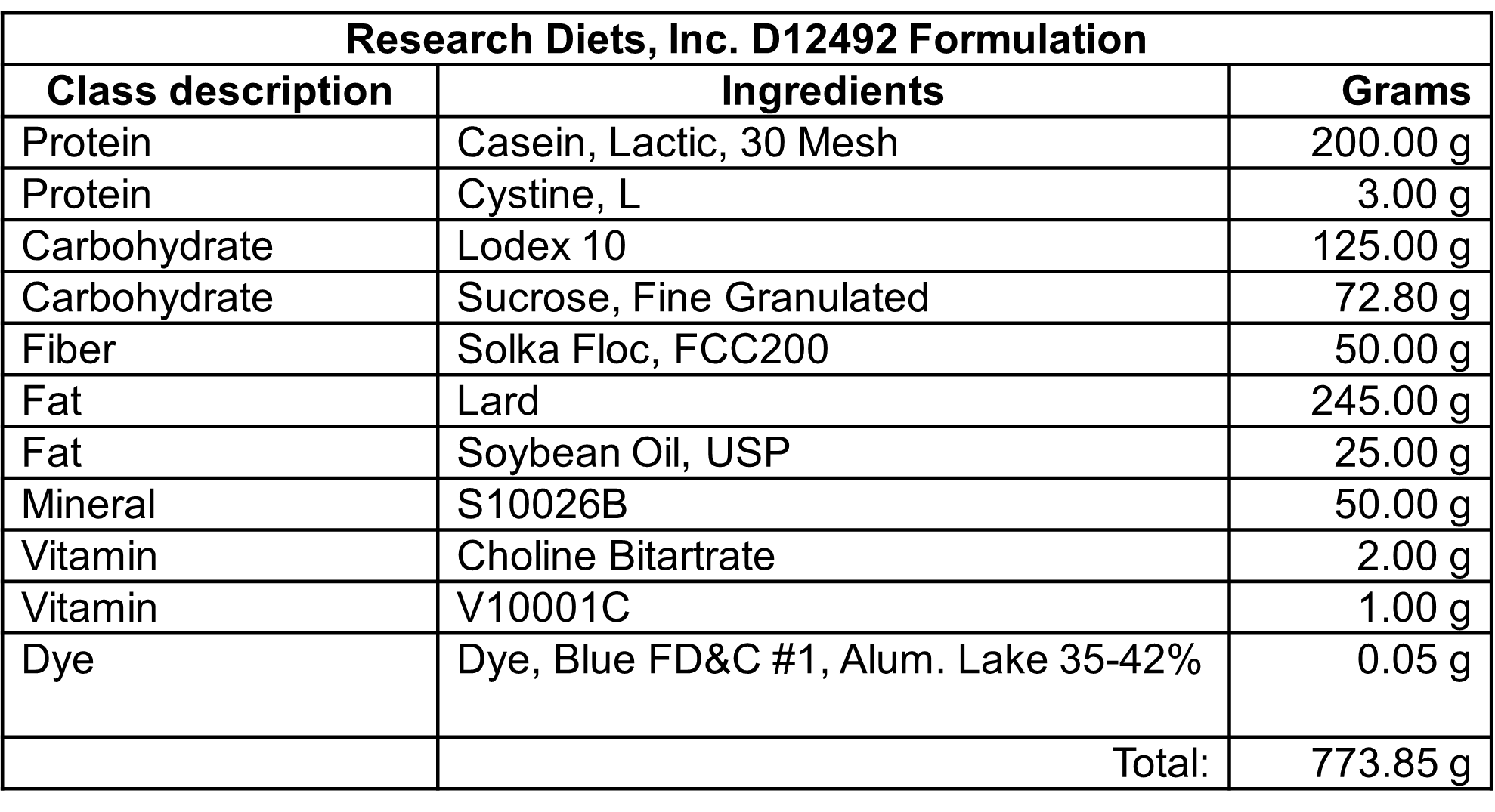

Supplement: S1 Table — Adapted from: https://researchdiets.com/formulas/d12492. (TIF) [file pone.0291347.s004.tif]
