# Supplementary material for: Daily replacement of very high-fat diet stabilizes food intake and improves mouse welfare by ensuring food quality
Source: PLoS One. 2023 Sep 18;18(9):e0291347. doi: 10.1371/journal.pone.0291347 (PMC10506720; doi:10.1371/journal.pone.0291347)
Supplement: Attachment — Submitted filename: Dear Authors R2.pdf [file pone.0291347.s008.pdf]

Dear Authors

Manuscript Number: PONE-D-23-21692 “Daily replacement of very high-fat diet stabilizes food intake and improves mouse welfare by ensuring food quality.

- The authors responded to my comments the Manuscript Number: PONE-D-23-21692 is accepted in my side .
