# Supplementary material for: Daily replacement of very high-fat diet stabilizes food intake and improves mouse welfare by ensuring food quality
Source: PLoS One. 2023 Sep 18;18(9):e0291347. doi: 10.1371/journal.pone.0291347 (PMC10506720; doi:10.1371/journal.pone.0291347)
Supplement: Attachment — Submitted filename: Authors PONE-D-23-21692.docx [file pone.0291347.s005.docx]

Dear Authors

Manuscript Number: PONE-D-23-21692 “Daily replacement of very high-fat diet stabilizes food intake and improves mouse welfare by ensuring food quality.

- The authors conducted experiments to establish best practices for ensuring the quality of VHFD and to improve mouse welfare. They found that VHFD in the food hopper was vulnerable first to dehydration and then oxidation within 7-days, leading to dramatic changes in food intake and food preference behavior in mice.
- This present study has not novel in its recommendations.

-	A separate part for the experiment design should be added to be clearer the manuscript.

-	This present study has not novel and the behavior study is not clear as to how the authors did it. The authors can use camera video to study the behaviors in mice.

-	The statistical tests used should be reported in the statistical analysis part.
