# Supplementary material for: Daily replacement of very high-fat diet stabilizes food intake and improves mouse welfare by ensuring food quality
Source: PLoS One. 2023 Sep 18;18(9):e0291347. doi: 10.1371/journal.pone.0291347 (PMC10506720; doi:10.1371/journal.pone.0291347)
Supplement: Attachment — Submitted filename: Response to reviewers.docx [file pone.0291347.s006.docx]

Response to Reviewers

**Journal requirements**:

**1.** **Please ensure that your manuscript meets PLOS ONE's style requirements, including those for file naming.**

I have ensured that the manuscript meets PLOS ONE’s style requirements.

**2. We note that you have stated that you will provide repository information for your data at acceptance. Should your manuscript be accepted for publication, we will hold it until you provide the relevant accession numbers or DOIs necessary to access your data. If you wish to make changes to your Data Availability statement, please describe these changes in your cover letter and we will update your Data Availability statement to reflect the information you provide.**

No changes to the Data Availability statement are required. The DOI to access data (<http://doi.org/10.6084/m9.figshare.23662566>) is already included.

**3.** **We note that you have referenced (unpublished) on page 11 which has currently not yet been accepted for publication. Please remove this from your References and amend this to state in the body of your manuscript: (ie “Bewick et al. [Unpublished]”) as detailed online in our guide for authors.**

“Cordeira et al. [Unpublished]” has been added to the body of the manuscript on page 11. The citation is excluded from References.

**Reviewer comments:**

Reviewer #1:

**The present study offers new and sound insights on the laboratory animal nutrition and welfare which will have a great impact on further upcoming studies. In my opinion graphical illustrations will make the manuscript more attractive to readership.**

Thank you for the wonderful suggestion! We revised Fig1, Fig2, Fig4, and S2Fig to include illustrations which we hope will make the manuscript more attractive.

Reviewer #2:

**-This present study has not novel in its recommendations.**

We are not the first to caution researchers about ensuring the quality of high-fat diets. However, methods used by researchers to ensure the quality of high-fat diets are underreported, variable, and unvalidated. Our study is the first to document pitfalls and welfare concerns which can specifically be avoided by daily replacement of a very high-fat diet.

**-A separate part for the experiment design should be added to be clearer the manuscript.**

Thank you for this suggestion. A new section entitled “Experimental design” has been added to the Methods to improve clarity.

**-This present study has not novel and the behavior study is not clear as to how the authors did it. The authors can use camera video to study the behaviors in mice.**

We hope the new experimental design section clarifies our methods for studying behavior. Yes, video cameras were used to study anxiety-like behavior in the open field test. We improved our word choice in the Results section to be clearer about this. Full details are described in the Methods.

**-The statistical tests used should be reported in the statistical analysis part.**

The statistical analysis section of the Methods has been revised to report and describe all statistical tests used.
